# Validation of the VALue of UteruS (VALUS) instrument in brazilian portuguese language for women with uterine fibroids and endometrial polyps: a cross-sectional study

**DOI:** 10.61622/rbgo/2026rbgo20

**Published:** 2026-05-12

**Authors:** Fernanda Rios Valvano, Carolina Neves Buhl, Glaucia Miranda Varella Pereira, Olivia Hui-Chiu Chang, Joao Paulo Leonardo-Pinto, Luiz Gustavo Oliveira Brito

**Affiliations:** 1 Universidade Estadual de Campinas Faculdade de Ciências Médicas Departmento de Tocoginecologia Campinas SP Brazil Departmento de Tocoginecologia, Faculdade de Ciências Médicas, Universidade Estadual de Campinas, Campinas, SP, Brazil.; 2 University of California Irvine School of Medicine Department of Urology Orange CA USA Department of Urology, School of Medicine, University of California Irvine, Orange, CA, USA.

**Keywords:** Hysterectomy, Uterus, Uterine myomectomy, Leiomyoma, Endometrial polyps, Visual Analog Scale, Psychometrics

## Abstract

**Objective:**

To translate and culturally validate the VALue of the UteruS (VALUS) instrument in Brazilian Portuguese language and to identify predictors for VALUS score.

**Methods:**

A cross-sectional study (February to November 2024) at a tertiary, academic hospital was performed with 164 women presenting symptomatic uterine fibroids (UF) (n=139) or endometrial polyps (n=25) that underwent hysterectomy or myomectomy/polypectomy. The VALUS instrument was translated and culturally validated first. Psychometric variables (internal consistency, test-retest, discriminant validity) were calculated. Women completed the VALUS instrument prior to the decision-making process to undergo surgery. The Uterine Fibroid Symptoms-Quality of Life (UFS-QOL) was also completed for women with UF. Uni- and multivariate linear regression was calculated to seek for associated factors with the VALUS total score.

**Results:**

Women that underwent hysterectomy were older, with higher education, higher parity and frequency of abdominal/pelvic surgeries than the non-hysterectomy group. VALUS scores were statistically higher in the non-hysterectomy for some questions and total score. Internal consistency of the VALUS instrument was excellent (alpha=0.890) and test-retest analysis did not differ between the groups (p=0.891). The UFS-QoL quality of life score presented a negative correlation with VALUS instrument. Multivariate linear regression demonstrated that the presence of abdominal/pelvic surgeries was associated with a lower VALUS score.

**Conclusion:**

VALUS demonstrated adequate internal consistency and reliability, but with low discriminant validity for the prediction of hysterectomy versus uterine-preservation in women with UF or endometrial polyps. Presence of abdominal/pelvic surgery is associated with a lower VALUS score.

## Introduction

Hysterectomy is one of the most frequently performed surgeries on women.^(1)^ As a definitive treatment for many gynecological disorders, it can be also considered a primary treatment. However, there has been a decrease in the number of procedures in the United States,^(1)^ Brazil,^(2)^ and probably one of the reasons for that is the growing availability of non-surgical or minimally invasive surgical options allowing for uterine-preservation.

Age can be an important variable for deciding whether removing or not the uterus, as the uterus is necessary for reproduction. However, it has been observed that even amongst older women with pelvic organ prolapse (POP), women may still choose to proceed with uterine preservation even if reproduction is not intended. Similarly, women with uterine fibroids beyond reproductive age may also choose to undergo myomectomy or non-surgical treatment than hysterectomy. Moreover, it is increasingly clear that women place value on the uterus beyond reproduction, and this may impact the type of surgical treatment for gynecologic conditions that women choose as we notice an increase of the hysteropexy rates.^(3)^

There are few, objective tools that measure the value for removing or not the uterus from the women´s standpoint. Recently, an objective instrument named Value of the Uterus (VALUS) was validated for women with and without POP over 45 years and presented good psychometric variables.^(4)^ It seems to be a more complete approach than asking if women wants to maintain their uterus because the questionnaire analyses other reasons for uterine preservation such as sense of self, femininity, relationship. Later, the same tool was tested in a cross-sectional study for other gynecological disorders but used the same cut-off point found for women with POP. They found that women who valued the uterus were highly motivated to be sexually active.^(5)^ There are opportunities to explore the use of the VALUS instrument within various gynecological disorders.

Uterine fibroids are the most common female pelvic tumor and can manifest with abnormal uterine bleeding and pelvic pain as frequent symptoms in 20-30% of women.^(6)^ Endometrial polyps are less common in younger women, but may be symptomatic as well.^(7)^ With this study, we validated the instrument for this population and compared it with other specific instruments, such as the Uterine Fibroid Symptoms Quality of Life (UFS-QOL) for women with uterine fibroids. We then aimed to calculate a cut-off point for the VALUS instrument for women with uterine fibroids or endometrial polyps, as well as to predictive factors associated with uterine preservation.

## Methods

### Study design, participants and inclusion/exclusion criteria

A cross-sectional study was conducted between February and November 2024 in a tertiary, university hospital in Brazil. We followed the STROBE guidelines for cross-sectional studies^(8)^ and the methodology of the validation study was based on Guidelines for Reporting Reliability and Agreement Studies (GRRAS).^(9)^

Women with symptomatic uterine fibroids or endometrial polyps that sought treatment were invited by medical residents or the medical research team to participate in the study, after an explanation of all study steps, during their first consultation at the clinic, prior to the decision-making process to undergo surgery. After they agreed to be enrolled at the study, they signed an informed consent and were given two questionnaires: one with sociodemographic variables and the VALUS instrument. For women with uterine fibroids, a third instrument (UFS-QOL) was also provided.

We excluded postmenopausal women due to higher risk for malignancy secondary to abnormal uterine bleeding (AUB), active pregnancy, AUB for non-structural causes or by adenomyosis or with gynecological premalignant cervical lesions or gynecological neoplasms.

Of the 800 eligible women seen in the outpatient clinic, 164 agreed to participate and were enrolled on the study. Of these women, 22 were randomly selected to fill out the questionnaires again after two weeks for the test-retest analysis.

### Variables, VALUS instrument, UFS-QOL questionnaire and Statistical analysis

**Figure 1:**
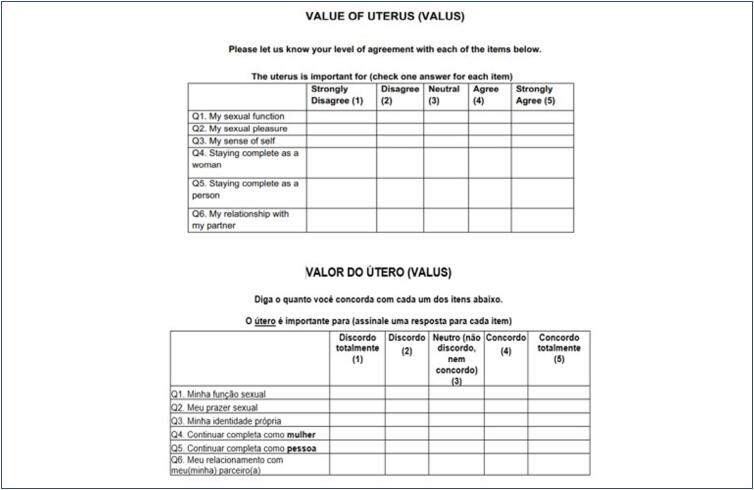
VALUS instrument in English and Brazilian Portuguese language

The VALUS questionnaire is a tool consisting of six questions related to the importance of the uterus for women and the impacts of its removal. Each question has five response options: completely disagree, disagree, neutral, agree, and strongly agree. The scoring is based on the sum of the responses, where one corresponds to "completely disagree" and five to "strongly agree," (score range: 6-30). After obtaining consent for validating the questionnaire by the corresponding authors (O.H.C), translation from English to Portuguese was performed by a notarized professional translator who was not aware of the objectives of the study and then adapted by three medical specialists (F.R.Valvano, J.P.Leonardo-Pinto, L.G.O.Brito). Subsequently, the translation from Brazilian Portuguese into English was performed (back translation) by a translator who was not aware of the original version of the questionnaire. This initial version was tested in a pilot phase of the study including 10 participants, who judged whether the items and expressions were comprehensible through cognitive interviews. We did not assess the Content Validity Index (CVI) because we thought this was not necessary as we did several meetings after the pilot phase of the questionnaire to assess whether all interviewers were carrying on the interviews on the same manner. After that, the instrument was finally finished, and we obtained a final version for application. Figure 1 displays the original and the validated version.

Based on the original study, the higher the score the likelihood of placing greater value on the uterus. The cutoff score ≥ 14 was calculated for women with pelvic organ prolapse. As we are studying women with UF and endometrial polyps, participants were grouped into those who underwent hysterectomy versus uterine-preservation. The VALUS cut-off score was identified to determine whether participants will choose hysterectomy or uterine-preservation based on the total VALUS score.

Additional data obtained included sociodemographic variables such as age (years), ethnicity (self-declared white or non-white), years of education (years of education informed by the participants), occupation (work activities informed by the participants) and clinical variables (height (meters), weight (kilos), body mass index, previous abdominal/pelvic surgeries (any surgery performed in the abdominal or pelvic region reported by the participant), presence of sexual activity (yes/no), comorbidities (hypertension/diabetes mellitus) and medications (used by the participants during the period of the study).

The UFS-QOL questionnaire (Uterine Fibroid Symptoms and Quality of Life) is a validated tool in Portuguese that includes eight questions evaluating the severity of symptoms in women with leiomyomas and 29 questions assessing their quality of life. The quality-of-life assessment is divided into subsections: concern, activities, energy/mood, control, self-consciousness, and sexual function. All questions offer five response options: "nothing," "very little," "a little," "a lot," "very much," or "never," "rarely," "sometimes," "most of the time," and "all the time," with values ranging from 1 to 5, where 1 corresponds to "nothing/never" and 5 to "very much/all the time." The higher the score, the greater the symptom severity, and the worse the quality of life.^(10)^

To describe the sample profile according to the variables under study, frequency tables were created for the categorical variables (surgery, ethnicity, etc.), with absolute frequency (n) and percentage (%) values, and descriptive statistics for the numerical variables (age, BMI, etc.), including mean, standard deviation, minimum and maximum values. Regarding missing data, no questionnaires presented more than 50% of incomplete data. We did not use any imputation methods. Based on previous publications,^(5)^ a minimum sample size of 132 participants would be need to complete the survey.

To compare categorical variables between the two groups (hysterectomy and non-hysterectomy), the Chi-Square or Fisher's exact tests (for expected values less than 5) were used. For continuous variables, the Student t test was performed. To study the relationship between numerical variables, Pearson's correlation coefficient was calculated. Construct validity was assessed via the calculation of correlation between the UFS-QOL severity and quality of life scores and the VALUS total score

To assess the internal consistency of the VALUS questionnaire, Cronbach's alpha coefficient was calculated for each question and total scores. To evaluate temporal stability (test-retest), the intraclass correlation coefficient (ICC) was computed. Paired Student-t test for related samples was used to compare VALUS scores between test and retest.

To study the factors related to the categorized VALUS score, simple and multiple linear regression analyses were conducted with stepwise with backward elimination technique with VALUS total score as continuous outcome. The significance level adopted for statistical tests was 5%, i.e., p < 0.05. Statistical analysis was performed by Intercooled Stata version 18.0 (College Station, TX, USA) and SAS version 9.4 (Cary, NC. USA). The study protocol was approved by the Institutional Review Board (CAAE 74699323.8.0000.5404, approval no 6.478.674).

## Results

Of the 164 participants, 139 presented uterine fibroids and 25 endometrial polyps. Sixty-eight underwent myomectomy/ polypectomy (non-hysterectomy group) and 96 underwent hysterectomy (hysterectomy group). Patient characteristics are displayed in table 1. Mean age was 43.29±6.77 years, ranging from 22 to 54. Women from the hysterectomy group were nine years older (p<0.001) than women from the non-hysterectomy group, with more years of education (p=0.042), higher parity (p=0.008) and higher history of abdominal/pelvic surgeries (p=0.012). Most of participants considered non-white (59.14%). No differences were noted between groups regarding ethnicity, BMI, presence of sexual activity, comorbidities.

**Table 1:** Sociodemographic and clinical variables between women that underwent hysterectomy or non-hysterectomy (myomectomy or polypectomy) procedures

Variables		Hysterectomy (n=96)		Non-hysterectomy (n = 68)		p-value
		n(%)	Mean ± SD	n(%)	Mean ± SD	
Age (Years)			45.76 ± 4.53		36.69 ±7.80	<0.001*
Ethnicity	White	37(38.54)		30(44.12)		0.474
	Non-white	59(61.46)		38(55.88)		
Years of education			13.07 ± 4.37		11.55 ± 4.51	0.042*
Number of pregnancies, birth and abortions	Pregnancy		2.21± 1.65		1.68 ± 1.89	0.008*
	Vaginal delivery		0.75 ± 1.15		0.76 ± 1.57	0.429
	Cesarean section		1.09 ± 1.22		0.63 ± 1.01	0.004*
	Abortions		0.36 ± 0.76		0.28 ± 0.67	0.442
Weight (kilos)			79.20 ± 17.76		80.81 ± 16.65	0.266
Height (meters)			1.63 ± 0.07		1.62 ± 0.06	0.295
BMI (KG/M2)			30.17 ± 5.69		29.93 ± 6.29	0.545
Abdominal/pelvic surgery	Yes	73(76.84)		39(58.21)		0.012**
	No	22(23.16)		28(41.79)		
Sexual activity	Yes	72(75)		57(83.82)		0.174
	No	24(25)		11(16.18)		
Comorbidities	Yes	59(61.46)		36(52.94)		0.276
	No	37(38.54)		32(47.06)		

* t-Student test;

** chi-square test; bold p-values are statistically significant

Table 2 displays the analysis of discriminant validity between the VALUS scores for each question and total score between groups. Participants from the hysterectomy group presented, in general, lower scores than women from the non-hysterectomy group, with statistically significant difference on questions 1 (p=0.007), 2 (p=0.023) and total score (p=0.036).

**Table 2:** Discriminant validity of the VALUS instrument between women who underwent hysterectomy or non-hysterectomy (myomectomy or polypectomy) procedures

	Hysterectomy (n=96)	Non-hysterectomy (n = 68)	p-value*
	Mean (± SD)	Mean (± SD)	
VALUS Q1	2.22 ± 1.26	2.76 ± 1.32	0.007
VALUS Q2	2.18 ± 1.21	2.62 ± 1.32	0.023
VALUS Q3	2.36 ± 1.38	2.78 ± 1.41	0.063
VALUS Q4	2.45 ± 1.39	2.66 ± 1.45	0.380
VALUS Q5	2.38 ± 1.42	2.53 ± 1.46	0.520
VALUS Q6	2.18 ± 1.26	2.43 ± 1.29	0.181
Total Score	13.76 ± 6.49	15.78 ± 6.40	0.036

* t-Student test; bold p-values are statistically significant

**Figure 2:**
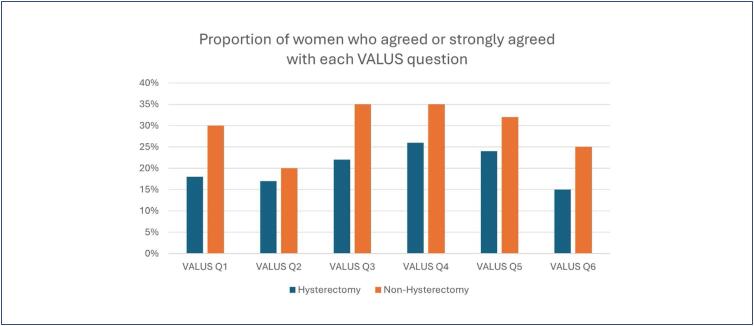
Women who agreed or strongly agreed according to VALUS questions divided by surgical procedures (hysterectomy or myomectomy/polypectomy)

Figure 2 shows the proportion of women who agreed or strongly agreed with each VALUS question. The non-hysterectomy group presented higher proportion of agreed/strongly agreed than the hysterectomy group; but this was not statistically significant.

Internal consistency of the VALUS total score was 0.890 and Cronbach´s alpha for retest was higher (0.943). After dividing per groups (Table 3), mean alpha for the hysterectomy and non-hysterectomy groups were respectively, 0.802 and 0.867, demonstrating excellent concordance. Test-retest analysis of the VALUS questionnaire did not differ between groups (p=0.891) with good concordance (ICC=0.679[0,365-0.853]; p<0.001).

**Table 3:** Internal consistency (Cronbach´s alpha) with item-test, item-rest and co-variance of the VALUS instrument between women who underwent hysterectomy or non-hysterectomy (myomectomy or polypectomy) procedures

	Hysterectomy (n = 96)				Non-hysterectomy			
	Item-test	Item-rest	Co-variance	Alpha	(n = 68)	Item rest	Co-variance	Alpha
VALUSQ1	0.727	0.676	3.174	0.781	0.693	0.633	3.056	0.773
VALUSQ2	0.819	0.785	3.102	0.774	0.764	0.715	2.976	0.766
VALUSQ3	0.784	0.738	3.052	0.772	0.753	0.699	2.943	0.764
VALUSQ4	0.849	0.815	2.967	0.764	0.777	0.725	2.897	0.760
VALUSQ5	0.861	0.828	2.939	0.762	0.810	0.765	2.850	0.756
VALUSQ6	0.872	0.845	3.013	0.767	0.860	0.831	2.885	0.756
Mean				0.802				0.867

Within women with uterine fibroids, no correlation was observed between severity of symptoms measured by UFS-QOL and total VALUS score (r=-0.11; p=0.22); a negative correlation was observed between the quality of life measured by UFS-QOL and total VALUS score (r=-0.21; p=0.01). Supplementary Material - Table 1S shows the UFS-QOL score for each question and their domains and most of the data did not show statistical significance, although all means from the hysterectomy group were higher than the myomectomy group. Univariate and multivariable logistic regression was performed to seek associated factors with the VALUS instrument (Table 4). The only remaining factor was previous abdominal/pelvic surgery, which meant that, when present, the lowest the VALUS score was found for these women (beta coefficient -3.2[-5.5 to 0.8];p=0.007).

**Table 4:** Univariate and multivariate linear regression for associate predictors with total VALUS score

Independent variable	Crude beta (95% CI)	p-value	Adjusted beta* (95% CI)	p-value
Age	-0.101(-0.249 to 0.047)	0.180	-0.040(-0.209 to 0.129)	0.639
Ethnicity	-0.139(-1.236 to 0.958)	0.803		
Years of education	0.103(-0.122 to 0.329)	0.368		
BMI	-0.131(-0.303 to 0.040)	0.132	-0.091(-0.265 to 0.083)	0.304
Parity	-0.611(-1.174 to -0.048)	0.034	-0.327(-0.968 to 0.313)	0.314
Previous abdominal/pelvic surgery	-3.854(-5.952 to -1.755)	<0.001	-3.202(-5.527 to -0.877)	0.007
Presence of sexual activity	0.214 (-2.242 to 2.672)	0.863		
Comorbidities	-1.770(-3.791 to 0.250)	0.086	-0.955(-3.073 to 1.161)	0.374

* included only variables whose p-value <0.20 for the final model

## Discussion

The translation and cultural validation of the VALUS instrument was successful to Brazilian Portuguese language by presenting adequate internal consistency, construct validity and test-retest. Discriminant validity between hysterectomy and non-hysterectomy groups were present on questions 1, 2 and total score and this correlation cannot be considered strong, although we think that a difference can be seen between groups as all means from all questions of the hysterectomy were lower than the non-hysterectomy group. Thus, it is important to consider a confirmatory factorial analysis in future studies as we need to clarify other measurements for discriminant validity of this instrument.

This study has found that women presenting UF or endometrial polyps with history of previous abdominal and/or pelvic surgery place less value on their uterus. There is no published study analyzing this independent variable, but it is possible to consider that women with a surgical history may be less averse to surgery. Visually, we can see higher means on Figure 2 by the proportion of agreed/strongly agreed from women that underwent hysterectomy, but this was not sufficient to find a statistically significant difference.

The strength of this study is that it presented a larger number of participants, included women with a broader age range, and used another questionnaire to analyze construct validity. The VALUS instrument was carefully translated and culturally validated in order to reduce response or language bias, and psychometric variables related to the questionnaire per se presented similar results to the original publication. Limitations are related to the study design itself (cross-sectional and the impossibility to stablish causal relationship), data collection was performed in a tertiary large system, different from primary health units, limiting generalizability; selection bias as this might be considered a survey. Another challenge was that many of these participants were already preconditioned to undergo one or other option for surgery, as tertiary hospitals are often the place where they are operated and these might be motivating factors, as published previously -Different from previous studies assessing this instrument, we presented a larger number of participants with UF or endometrial polyps and although age was not a confounding factor in the VALUS score, our range did not include postmenopausal women, and perhaps discriminant validity may have not been properly analyzed due to this situation.

Comparison with available literature is difficult to make because there is scant data on the use of objective tools to measure beliefs of women to choose one or another treatment option for their gynecological disorder. Our data concerning discriminant validity conflicts with the first study that targeted women with pelvic organ prolapse, on which there is a clear difference between the mean scores from VALUS questions. As post-menopausal women were excluded from this study, the mean age was twenty years younger than the primary study. Since the original validation study excluded women who desired future pregnancy, we believe that this may be the confounder as to why VALUS was not able to discriminate between those who choose hysterectomy versus uterine-preservation in this study. The VALUS instrument does not evaluate desire for pregnancy or fertility in the valuation of the uterus.

The importance of documenting the beliefs and reasons for valuation of uterus is that there is a growing interest to not removing it and that literature has been rediscussing the need for hysterectomy in cases that were common to remove it such as POP.^(11)^ Moreover, it is known that benign hysterectomy may have long-term effects. A recent systematic review has demonstrated that hysterectomy with bilateral salpingo-oophorectomy in younger women increases the risk for colorectal cancer, total cardiovascular disease, and coronary heart diseases.^(12)^ Furthermore, there is growing evidence that hysteropexy may be non-inferior or equivalent to hysterectomy for women with POP in non-randomized^(13)^ and randomized studies.^(14)^

For women who have access to and present the option to choose between hysterectomy and myomectomy/polypectomy, decision-making is complex and involves both patient preferences and physician counseling. This shared decision model includes risk and benefits of both options, discussion of the preoperative informed consent. Placing value for the uterus is a multidimensional process that includes reproductive, sexual and identity-related aspects and this should be considered with future confirmatory or exploratory factor analysis within future studies. Further large-scale studies are needed to explore whether this instrument behaves differently with women with UF or endometrial polyps than with POP.

## Conclusion

The cultural translation and validation of the VALUS questionnaire demonstrated adequate internal consistency and reliability, but low discriminant validity for the prediction of hysterectomy versus uterine-preservation in women with UF or endometrial polyps. Presence of abdominal/pelvic surgery is associated with a lower VALUS score.

## Data Availability

The research data are described in the article presented.
