# Supplementary material for: Validation of the VALue of UteruS (VALUS) instrument in brazilian portuguese language for women with uterine fibroids and endometrial polyps: a cross-sectional study
Source: Rev Bras Ginecol Obstet. 2026 May 12;48:e-rbgo20. doi: 10.61622/rbgo/2026rbgo20 (PMC13399368; doi:10.61622/rbgo/2026rbgo20)
Supplement: Supplementary Material [file 1806-9339-rbgo-48-e-rbgo20-Supp01.pdf]

Supplementary Material

Table 1S. UFS-QOL mean scores between women with uterine fibroids who underwent hysterectomy or myomectomy

	Hysterectomy<br>Mean (± SD)	Myomectomy<br>Mean (± SD)	p-value*
UFSQOL Q1	5.65±9.50	4.27±1.14	0.379
UFSQOL Q2	4.22±0.93	3.91±1.08	0.136
UFSQOL Q3	4.10±1.13	3.89±1.28	0.384
UFSQOL Q4	3.92±1.07	3.52±1.28	0.098
UFSQOL Q5	4.19±0.84	4.10±0.96	0.627
UFSQOL Q6	3.97±1.24	3.59±1.27	0.147
UFSQOL Q7	3.83±1.23	3.37±1.40	0.088
Severity Score	32.65±12.58	27.56±11.06	0.033
UFSQOL Q8	4.38±0.86	3.94±1.15	0.029
UFSQOL Q9	3.66±1.21	3.37±1.29	0.263
UFSQOL Q10	3.19±1.46	3.08±1.51	0.711
UFSQOL Q11	3.68±1.33	3.66±1.37	0.956
UFSQOL Q12	3.98±1.13	3.59±1.23	0.107
UFSQOL Q13	3.56±1.27	3.35±1.39	0.442
UFSQOL Q14	3.72±1.31	3.05±1.56	0.021
UFSQOL Q15	4.11±1.25	3.59±1.40	0.052
UFSQOL Q16	3.70±1.38	3.45±1.40	0.397
UFSQOL Q17	3.43±1.26	3.08±1.08	0.158
UFSQOL Q18	3.50±1.39	3.21±1.52	0.327
UFSQOL Q19	3.76±2.28	2.91±1.32	0.002
UFSQOL Q20	3.60±1.26	2.83±1.29	0.004
UFSQOL Q21	4.07±1.27	3.83±1.32	0.372
UFSQOL Q22	4.16±1.14	3.83±1.50	0.214
UFSQOL Q23	4±1.18	3.58±1.40	0.113
UFSQOL Q24	3.77±1.19	3.32±1.33	0.079
UFSQOL Q25	3.83±1.21	3. ±1.33	0.369
UFSQOL Q26	4.28±0.91	3.81±1.12	0.022
UFSQOL Q27	3.95±1.07	3.48±1.30	0.051
UFSQOL Q28	4.05±1.30	4.72±4.80	0.284
UFSQOL Q29	3.62±1.35	3.16±1.48	0.108
UFSQOL Q30	3.22±1.46	3.11±1.48	0.711
UFSQOL Q31	3.77±1.27	3.64±1.25	0.624
UFSQOL Q32	3.85±1.29	3.63±1.29	0.429
UFSQOL Q33	3.56±1.52	3.29±1.52	0.390
UFSQOL Q34	3.51±1.39	3.14±1.39	0.205
UFSQOL Q35	4±1.34	3.66±1.41	0.243
UFSQOL Q36	3.65±1.35	3.36±1.55	0.328
UFSQOL Q37	3.59±1.38	3.41±1.48	0.539
Score Quality of Life	80.63±52.05	74.40±49.51	0.494
